# Réactivation d’une hépatite B occulte chez un patient drépanocytaire homozygote: cas clinique et revue de la litérature

**DOI:** 10.11604/pamj.2017.28.127.13640

**Published:** 2017-10-10

**Authors:** Moustapha Diop, Viviane Marie Pierre Cisse-Diallo, Daye Ka, Ndèye Aissatou Lakhe, Khardiata Diallo-Mbaye, Aminata Massaly, Alassane Dièye, Ndèye Maguette Fall, Aboubacar Sadikh Badiane, Daouda Thioub, Louise Fortes-Déguénonvo, Gora Lo, Cheikh Tacko Diop, Cheikh Tidiane Ndour, Masserigne Soumaré, Moussa Seydi

**Affiliations:** 1 Service des Maladies Infectieuses et Tropicales, CHNU de Fann, Dakar, Sénégal; 2 Centre Médical Inter Armée Sud, Dakar, Sénégal; 3 Centre Hospitalier National Universitaire de Fann, Dakar, Sénégal

**Keywords:** Hépatite B occulte, réactivation, drépanocytose homozygote, Occult Hepatitis B, reactivation, homozygous sickle cell disease

## Abstract

L'hépatite B occulte correspond à la présence de l'ADN du virus de l'hépatite B dans le sérum et/ou dans le foie d'un patient malgré la négativité de l'AgHBs. C'est une forme clinique habituellement asymptomatique. Sa réactivation est rare et survient en général chez le sujet immunodéprimé. Nous rapportons un cas d'un patient sénégalais de 21 ans, drépanocytaire homozygote, qui présentait un ictère de type cholestatique chez qui l'exploration biologique concluait a une réactivation d'une hépatite B occulte. Cette observation souligne la nécessité de rechercher systématiquement une réactivation d'une hépatite B occulte devant toute hépatopathie aigue chez le drépanocytaire.

## Abstract (en)

Occult Hepatitis B corresponds to the presence of hepatitis B virus-deoxyribonucleic acid (HBV-DNA) in serum and/or in liver of a patient despite HBsAg negativity. Clinically, it is usually asymptomatic. Its reactivation is rare and commonly occurs in immunosuppressed individuals. We report the case of a 21-year old patient from Senegal, with homozygous sickle cell disease, presenting with cholestatic jaundice. Laboratory tests showed reactivation of occult Hepatitis B. This study emphasizes the need to systematically investigate the presence of occult Hepatitis B in patients with sickle cell disease suffering from acute liver disease.

## Introduction

L´hépatite B constitue un problème mondial de santé publique. Le nombre de personnes souffrant d´une infection chronique par le virus de l´hépatite B (VHB) est estimé à 240 millions parmi lesquelles 650000 meurent chaque année des complications de cette infection. L'Afrique sub-saharienne fait partie de la zone de forte prévalence selon l'OMS [1]. Au Sénégal, la prévalence du portage chronique de l'AgHbs est estimée à 11 % [2]. L'hépatite B occulte est une forme clinique de cette infection décrite au début des années 1980. Elle correspond à la présence de l'ADN du VHB dans le sérum et / ou dans le foie d'un patient chez qui l'AgHbs est indétectable dans le sérum par les tests sérologiques usuels [3]. Elle est habituellement asymptomatique et est caractérisée par un taux très faible de l'ADN du VHB. Cependant de rares cas de réactivations de l'hépatite B occulte ont été décrits dans la littérature sur des terrains d'immunodépression tels que : le traitement immunosuppresseur [4] et l'infection à VIH [5]. Nous rapportons un cas de réactivation d'une hépatite B occulte chez un patient sénégalais drépanocytaire homozygote.

## Patient et observation

**Figure 1:**
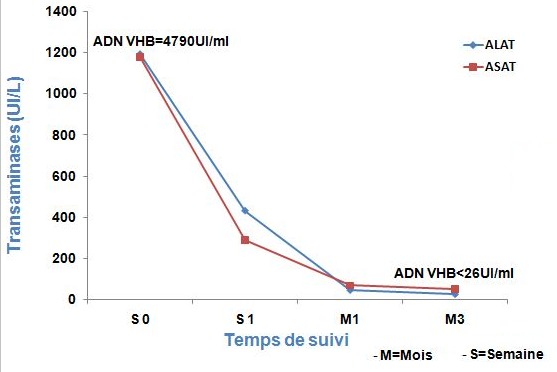
Evolution des transaminases et de l’ADN du VHB en fonction du temps

Il s'agissait d'un patient sénégalais âgé de 21 ans, drépanocytaire homozygote, hospitalisé au service des maladies infectieuses et tropicales du CHNU de Fann (Dakar) le 24 février 2017 pour des douleurs osseuses diffuses, une coloration jaunâtre des muqueuses conjonctivales et une fièvre d'allégation. Ce tableau clinique évoluait depuis 5 jours avant son admission. A l'examen clinique on notait une crise vaso-occlusive, un ictère de type cholestatique et une anémie clinique. Sur le plan biologique une cholestase était objectivée (Bilirubine totale à 54mg/dl (54N), Bilirubine conjugué à 28,8mg/dl (115N), PAL à 389,4UI/L (1,5N) et γ-GT à 280 UI/L (3N)) ainsi qu'une cytolyse hépatique (ALAT à 1192,4UI/L (29N), ASAT à 1179,8UI/L (28N)). La recherche d'AgHBs par deux tests qualitatifs (test de diagnostic rapide et Architect) était négative et sa quantification était aussi négative (<0,05UI/ml). Les dosages des anticorps anti-Hbc totaux, de la fraction IgM des anticorps anti-Hbc et de l'AgHbe étaient tous positifs et l'ADN virale était à 4790UI/ml. La recherche sérologique des virus de l'hépatite (C, D et E) était négative et celle du virus de l'hépatite A avait mis en évidence des anticorps anti-VHA de type IgG. La sérologie rétrovirale (VIH) était négative. L'hémogramme montrait une hyperleucocytose à 12.000 éléments/mm3 et une anémie à 10,2 g/dl normochrome, normocytaire et la protéine C réactive était à 25,76mg/. Sur le plan morphologique, l'échographie abdominale montrait une discrète splénomégalie sans hépatomégalie ni dysmorphie hépatique. Un traitement à base de sérum salé isotonique (1,5 litre / jour), de tramadol : 50 mg x 3 / jour en intra-veineuse (IV), d'ofloxacine : 200 mg x 2 / jour en IV pendant 10 jours et des mesures hygiéno-diététiques ont été instaurés. A une semaine d'hospitalisation, l'évolution était marquée par une apyrexie stable, une légère régression de l'ictère et une disparition des douleurs osseuses. A la biologique, la cholestase avait régressé avec un taux de Bilirubine totale à 18,8 mg/l (18N) et la bilirubine conjuguée à 12,7mg (51N). Les ALAT étaient à 431,8UI/L (10N) et les ASAT à 287,9 UI/L (7N). Le patient était sorti au huitième jour d'hospitalisation et était vacciné contre le pneumocoque et la fièvre typhoïde du fait de son terrain de drépanocytose homozygote. A un mois de suivi, les transaminases, les γ-GT et les phosphatases alcalines étaient normales. Les taux de bilirubine totale et conjuguée avaient régressé et étaient respectivement à 9,1mg/l (9N) et 3,8mg/l (15N). L'hémogramme de contrôle montrait une anémie à 10,3g/dl avec un taux de globules blancs normal. A trois mois de suivi, aucun signe clinique n'a été décelé, les transaminases étaient normales, la recherche d'AgHBe était négative, celle des anticorps anti-HBe était positive et l'ADN du VHB était devenu indétectable (<26UI/ml) (Figure 1). L'échographie abdominale était normale

## Discussion

La prévalence de l'hépatite B occulte en Afrique et au Sénégal en particulier n'est pas connue. Cependant quelques études réalisées sur des populations différentes rapportent des chiffres très variables. Ainsi, au Cameroun une prévalence de l'hépatite B occulte de 5,9% chez les patients vivant avec le VIH a été retrouvée [6] et ce taux était de 17,2% chez des donneurs de sang en Egypte [7]. De même, en Uganda, deux études réalisées chez les mêmes patients et utilisant des techniques de PCR différentes pour la quantification de la charge virale du VHB avaient trouvé des prévalences de 11,8% et 36% [8, 9]. Ces prévalences variables s'expliquent, entre autres, par l'hétérogénéité des populations d'étude mais aussi par les différences des tests sérologiques et des techniques de biologie moléculaire utilisés pour la détection respective de l'AgHbs et de l'ADN du VHB. La prévalence des hépatites B occultes est probablement sous-estimée dans nos régions. D'où de la nécessité de la dépister chez tout patient porteur d'une hépatopathie avec AgHbs négatif.

Même si la physiopathologie de l'hépatite B occulte n'est pas bien élucidée, plusieurs mécanismes sont évoqués. Il peut s'agir de la production d'une protéine S par le VHB antigéniquement modifiée et non détectable par les tests disponibles. Le virus peut aussi faire des mutations capables d'inhiber l'expression du gène S et/ou la réplication virale. Mais le plus souvent il s'agit d'une forte suppression de la réplication virale et de l'expression des gènes. Les facteurs pouvant induire ces mécanismes physiopathologiques sont, entre autres, la réponse immune, les co-infections avec d'autres agents infectieux et les facteurs épigénétiques [3]. L'hépatite B occulte est habituellement asymptomatique et est caractérisée par un taux de ADN viral faible (< 200UI/ ml) [3, 10]. Mais de rares cas de réactivations cliniques et biologiques de cette infection ont été décrits sur des terrains d'immunodépression tels que le traitement immunosuppresseur [4], l'infection à VIH [5] et la transplantation d'organes [11]. Chez notre patient la drépanocytose SS était probablement le terrain d'immunodépression favorisant la réactivation de l'hépatite B occulte. L'AgHBs est un marqueur très sensible de réactivation d'hépatite B occultes et revient positif dans la plupart des cas [3–5]. La particularité chez notre patient était la recherche d'AgHBs aussi bien par des tests qualitatifs que quantitatif négative.

Devant le syndrome de cholestase clinico-biologique, nous avons éliminé d'autres causes fréquentes d'hépatopathie pouvant être associées à la réactivation de l'hépatite B occulte. Notre patient n'avait pas pris une phytothérapie récente par voie orale, il ne prenait pas de l'alcool et il n'avait pas signalé de prise médicamenteuse. La recherche sérologique des autres hépatites virales C, D et E était négative. L'échographie abdominale montrait un foie de taille et d'échostructure normale et les voies biliaires étaient libres et non dilatées. Les anticorps anti-nucléaire, anti-LMK1, anti-muscle lisse et anti-mitochondrie n'ont pas été recherchés pour éliminer une hépatite auto-immune ou une cirrhose biliaire primitive. Mais ces deux affections étaient moins probables car notre patient est de sexe masculin et il n'avait pas de signes dysimmunitaires extrahépatique. Sur le plan thérapeutique, aucune recommandation n'est spécifique à la prise en de la forme occulte d'hépatite B. En se basant sur les directives actuelles de l'OMS [1], notre patient n'était pas éligible au traitement antivirale car son taux d'ADN virale était à 4790UI/ml (< 20.000UI/ml). Cependant, il est régulièrement suivi avec un monitoring des signes cliniques et biologiques d'hépatopathie.

## Conclusion

L'hépatite B occulte est une forme particulière de l'infection au VHB qui attire de plus en plus l'attention des cliniciens. Elle est habituellement asymptomatique, mais sa réactivation, bien que rare, reste possible sur un terrain d'immunodépression. Notre observation semble être le premier cas d'hépatite B occulte réactivée sur un terrain de drépanocytose publiée. Elle souligne la nécessité de rechercher systématiquement la réactivation d'une hépatite B occulte devant une hépaopathie aigue sur un terrain d'immunodépression comme la drépanocytose homozygote.

## Conflits d’intérêts

Les auteurs ne déclarent aucun conflit d'intérêts.
